# Neural Networks for Classification and Image Generation of Aging in Genetic Syndromes

**DOI:** 10.3389/fgene.2022.864092

**Published:** 2022-04-11

**Authors:** Dat Duong, Ping Hu, Cedrik Tekendo-Ngongang, Suzanna E. Ledgister Hanchard, Simon Liu, Benjamin D. Solomon, Rebekah L. Waikel

**Affiliations:** Medical Genomics Unit, National Human Genome Research Institute, Bethesda, MD, United States

**Keywords:** deep learning, generative adversarial networks, 22q11.2 deletion syndrome, aging, Williams syndrome, facial recognition, facial diagnosis

## Abstract

**Background:** In medical genetics, one application of neural networks is the diagnosis of genetic diseases based on images of patient faces. While these applications have been validated in the literature with primarily pediatric subjects, it is not known whether these applications can accurately diagnose patients across a lifespan. We aimed to extend previous works to determine whether age plays a factor in facial diagnosis as well as to explore other factors that may contribute to the overall diagnostic accuracy.

**Methods:** To investigate this, we chose two relatively common conditions, Williams syndrome and 22q11.2 deletion syndrome. We built a neural network classifier trained on images of affected and unaffected individuals of different ages and compared classifier accuracy to clinical geneticists. We analyzed the results of saliency maps and the use of generative adversarial networks to boost accuracy.

**Results:** Our classifier outperformed clinical geneticists at recognizing face images of these two conditions within each of the age groups (the performance varied between the age groups): 1) under 2 years old, 2) 2–9 years old, 3) 10–19 years old, 4) 20–34 years old, and 5) ≥35 years old. The overall accuracy improvement by our classifier over the clinical geneticists was 15.5 and 22.7% for Williams syndrome and 22q11.2 deletion syndrome, respectively. Additionally, comparison of saliency maps revealed that key facial features learned by the neural network differed with respect to age. Finally, joint training real images with multiple different types of fake images created by a generative adversarial network showed up to 3.25% accuracy gain in classification accuracy.

**Conclusion:** The ability of clinical geneticists to diagnose these conditions is influenced by the age of the patient. Deep learning technologies such as our classifier can more accurately identify patients across the lifespan based on facial features. Saliency maps of computer vision reveal that the syndromic facial feature attributes change with the age of the patient. Modest improvements in the classifier accuracy were observed when joint training was carried out with both real and fake images. Our findings highlight the need for a greater focus on age as a confounder in facial diagnosis.

## Background

Neural networks are emerging as powerful tools in many areas of biomedical research and are starting to impact clinical care. In the field of genomics, these methods are applied in multiple ways, including generating differential diagnoses for patients with a possible genetic syndrome based on images, (Gurovich et al., 2019; Hsieh et al., 2021; Porras et al., 2021), analysis of DNA sequencing data (Luo et al., 2019) including phenotype-based annotation (Clark et al., 2019) and variant classification (Frazer et al., 2021), and prediction of the protein structure (Baek et al., 2021; Jumper et al., 2021).

In the field of clinical genetics, clinicians typically encounter many different conditions that are individually rare and which can be difficult to differentiate (Solomon et al., 2013; Ferreira, 2019). This complexity, coupled with a lack of trained experts (Maiese et al., 2019; Jenkins et al., 2021), can lead to delayed diagnosis and suboptimal management for affected people (Gonzaludo et al., 2019). Such challenges can disproportionately impact older patients, as many clinical geneticists are initially trained in pediatric medicine and tend to focus on pediatric diagnosis (Jenkins et al., 2021). Despite these issues, previous large-scale clinical genetic applications of neural networks studied populations affected by many different genetic conditions and yielded impressive results (Gurovich et al., 2019; Porras et al., 2021). In the current study, we endeavored to build upon these existing works by collating our own age-annotated datasets. These datasets were designed to allow further study of the impact of patient age on facial diagnosis as well as to perform additional neural network analyses, which can also be extended to larger datasets or applied to different conditions.

We chose two distinct genetic conditions for further study: Williams syndrome (WS) (MIM 194050), which affects approximately one in 7,500 live births, and 22q11.2 deletion syndrome (22q), sometimes imperfectly referred to as “DiGeorge syndrome” (MIM 188400), which affects approximately one in 4,000–7,000 live births (Stromme et al., 2002; Botto et al., 2003; Oskarsdottir et al., 2004). We selected these conditions as they may be recognizable from facial features (in addition to other manifestations) (Stromme et al., 2002; Botto et al., 2003; Campbell et al., 2018; Morris et al., 2020) and based on relative data availability, which is still very limited compared to more common health conditions. Additionally, these two conditions represent varying ease of diagnosis based on facial appearances: people with WS may have more consistently recognizable facial features, whereas people with 22q may have a more subtle facial presentation, which likely contributes to underdiagnosis for this as well as many other conditions without obvious or overtly pathognomonic signs.

To examine the influence of age on facial recognition, we evaluated how well clinical geneticists and our classifier recognize these conditions based on facial images of varying ages. We further explored additional neural networks applications, including saliency maps and generative adversarial networks (GANs), to study both facial recognition as a whole and as a function of age.

## Materials and Methods

### Data Collection

**FIGURE 1:**
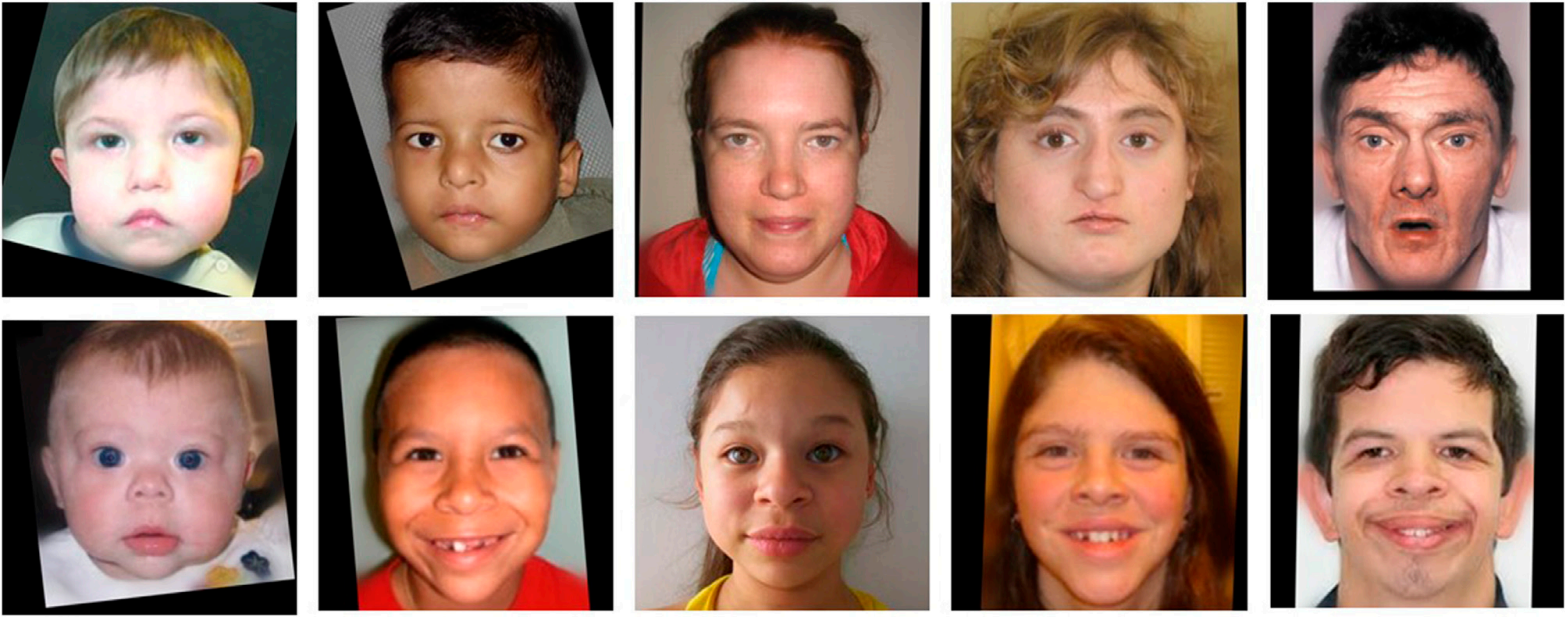
Centered and aligned images of real individuals (different individuals are shown at different ages) affected with 22q (top row) and WS (bottom row). These images have been previously published and are granted to be freely distributed for noncommercial research purposes.

We searched Google and PubMed using the disease names of interest to select publicly available images depicting individuals with WS, 22q, or other genetic conditions that may resemble WS or 22q (see Supplementary Table S1 for more details about these conditions). After that, when the context is clear, we refer to these “other genetic conditions” as the control group. From the available source information for each image, we categorized the images into five age brackets: 1) infant (under 2 years old), 2) child (2–9 years old), 3) adolescent (10–19 years old), 4) young adult (20–34 years old), and 5) older adult (≥35 years old). We attempted to collect images of individuals from diverse ancestral backgrounds, though standardized and complete information regarding race and ethnicity was often unavailable (see Supplementary Table S2). In total, we collected 1,894 images and partitioned them into 1,713 and 181 train and test images, respectively (see Supplementary Table S3). The image sets included both color and black and white images with varying image resolution. The test images were selected from color images subjectively judged to have adequate resolution for human viewing and included representations of both sexes and of apparently ancestrally diverse individuals, though we recognized many challenges in these and related areas (Byeon et al., 2021). The control group test images included individuals with other genetic and congenital conditions, including those with overlapping facial features with WS or 22q (i.e., conditions that are sometimes considered in the differential diagnosis of WS or 22q). We applied the StyleGAN face detector and image preprocessing to rotate and center our images and manually aligned images that failed this preprocessing step (Figure 1).

### Classifier

We selected the EfficientNet-B4 classifier, which obtained high performance on the ImageNet data with a relatively low number of parameters (Tan and Le, 2019). We loaded the weights pretrained on ImageNet and continued training EfficientNet-B4 end-to-end. Combining and then jointly training a small dataset of interest with a larger auxiliary dataset often increases the prediction accuracy (Ahmad et al., 2018; Meftah et al., 2020). Our auxiliary dataset is the FairFace dataset, which contains 108,000 “in-the-wild” faces (i.e., faces oriented in various angles and/or partially covered with hands, hats, or sunglasses) of equal ratio from (using definitions in FairFace) white, black, Latino, East/Southeast Asian, Indian, and Middle Eastern populations (Kärkkäinen and Joo, 2019).

The StyleGAN face detector and image preprocessing, such as rotating and centering faces, were applied to the in-the-wild FairFace faces, resulting in 62,088 usable images. We partitioned these 62,088 images into the age groups as described previously via the FairFace age classifier. One-sixth of the images (N = 10,348) in each age category were randomly chosen as test images, which we evaluated with our own test images. The remaining 51,740 images were used with our images to train EfficientNet-B4.

Because of our small dataset and the assumption that at least some features persisted across age groups, we trained EfficientNet-B4 on our images (regardless of age) and FairFace images, to recognize the four labels: WS, 22q, other genetic conditions (control), and unaffected. We included unaffected individuals as an important consideration in clinical practice, which is the ability to differentiate a potentially affected from an unaffected person, especially as some genetic conditions can have subtle findings often missed by general clinicians as well as subspecialists. The classifier was trained with cross entropy loss function in which one-hot encodings represent true image labels. We rescaled all the images into resolution 448 × 448 pixels when training EfficientNet-B4. Image resolution was chosen to maximize GPU usage (two Nvidia P100, training batch size 64).

We trained five classifiers via 5-fold cross-validation (one for each fold) and then created an ensemble predictor by averaging the predicted label probabilities of an image from these five classifiers. When averaging, we considered only the classifiers that produced a maximum predicted probability (over all the labels) of at least 0.5.

### Comparison to Clinicians

We compared our classifier to board-certified or board-eligible clinical geneticist physicians via surveys sent by Qualtrics (Provo, Utah, United States). As WS and 22q syndromes are relatively distinct, we felt that it was more meaningful to evaluate WS test images against their own controls and likewise for 22q test images. We emphasize that for nontrivial comparisons, the control test images were of conditions resembling WS and 22q. For WS surveys, there were 50 WS (10 images per age group) and 50 corresponding control test images. To keep the survey length reasonable, each participant went through a random subset of 25 WS (5 images per age category) and 25 age category-matched control images. The ordering of the selected images in a survey was randomized, and the answer choice for a question was either “Williams Syndrome” or “Other Condition.” The same setup was also employed for 22q surveys. In addition to asking clinical geneticists to classify images, we also asked questions about the impact of patients’ age on diagnosis to determine attitudes and opinions on the age in the diagnosis process. Example surveys can be found at https://github.com/datduong/Classify-WS-22q-Img.

Following previous methods (Tschandl et al., 2018; Tschandl et al., 2019; Duong et al., 2021b), we estimated that 30 participants would provide a statistical power of 95% to detect a 10% difference. The participants were recruited via email. To identify survey respondents, we obtained email addresses through professional networks, departmental websites, journal publications, and other web-available lists. A total of 225 clinical geneticists were contacted, of which 36 completed the 22q survey and 34 completed the WS survey. If multiple respondents completed the same survey, only the first survey was used for analysis (see Supplementary Table S4 for the description of survey respondents).

### Generative Adversarial Network (GAN)

We trained a GAN for each data partition from the 5-fold cross-validation in section “*Classifier*.*”* We describe the GAN training and image generation for a data partition *p*, which also will apply to the other partitions (see Supplementary Figure S1 for the flowchart of our GAN image production and its application with the real images to train the disease classifier). The partition *p* contains our images of affected individuals and FairFace unaffected individuals. Ideally, we would want to train the GAN model on all FairFace images and our dataset as blending the features of different racial/ethnic groups in FairFace with our dataset would generate diverse images of affected individuals. However, the partition *p* has approximately 41,392 images of unaffected FairFace individuals, and our preliminary GAN experiments required a large amount of computational power. The larger FairFace dataset also often skewed GAN output in which the generated images of affected individuals looked more like the unaffected subset. Therefore, in the partition *p*, we trained GAN on our images and a fixed subset of FairFace. This subset was randomly chosen with 500 individuals in each age bracket. In each training batch, we selected an equal number of affected and unaffected individuals.

Our GAN is based on the conditional StyleGAN2-ADA and generates images using both disease statuses and age categories (Karras et al., 2020). We made the following key modification to StyleGAN2-ADA. The default label embedding is L x 512, where L is the number of labels and produces a vector of length 512 for each label. Training this embedding requires many people with a specific disease in a certain age category. However, some disease and age label combinations have small sample sizes; for example, our dataset has 39 WS and 35 22q individuals older than 35 years of age. We replaced the default label embedding with two smaller matrices, namely 4 × 256 and 5 × 256 to represent the four diseases (WS, 22q, other conditions, and unaffected) and five age categories. Then, training the 4 × 256 disease embedding uses all affected individuals in every age category. Likewise, training the 5 × 256 age embedding uses all the images in our dataset and in FairFace. The outputs of these two components are concatenated to a vector of size 512 to match the rest of the StyleGAN2-ADA architecture. Hence, except for the label embeddings, we initialized all the StyleGAN2-ADA weights with the pretrained values on FFHQ dataset at resolution 256 × 256 pixels (Karras et al., 2020); all fake images were also generated in the resolution 256 × 256 pixels. Image resolution was chosen to maximize GPU usage (two Nvidia P100).

After training GAN on the data partition *p*, we generated four types of fake images: 1) unrelated faces for a specific disease and age group; 2) similar faces at different ages for a specific disease; 3) the same face at different ages for a specific disease; and 4) faces containing characteristics of two different conditions, which we hypothesized could aid classifier accuracy.

Given the large number of unaffected people in FairFace, we generated just images of affected individuals from the disease label d∈ {WS, 22q, other condition} and age bracket a∈ {infant, child, adolescent, young adult, older adult}. The number of generated images for each pair (*d,a*) is equal to the average count of all age groups with a specific disease *d* in the data partition. Thus, for a specific condition, respectively, we made more and fewer images for the uncommon (less represented) and common (more represented) age groups. In total, each image type has the same count as the size of the affected individuals in the data partition in which the GAN was trained.

**FIGURE 2:**
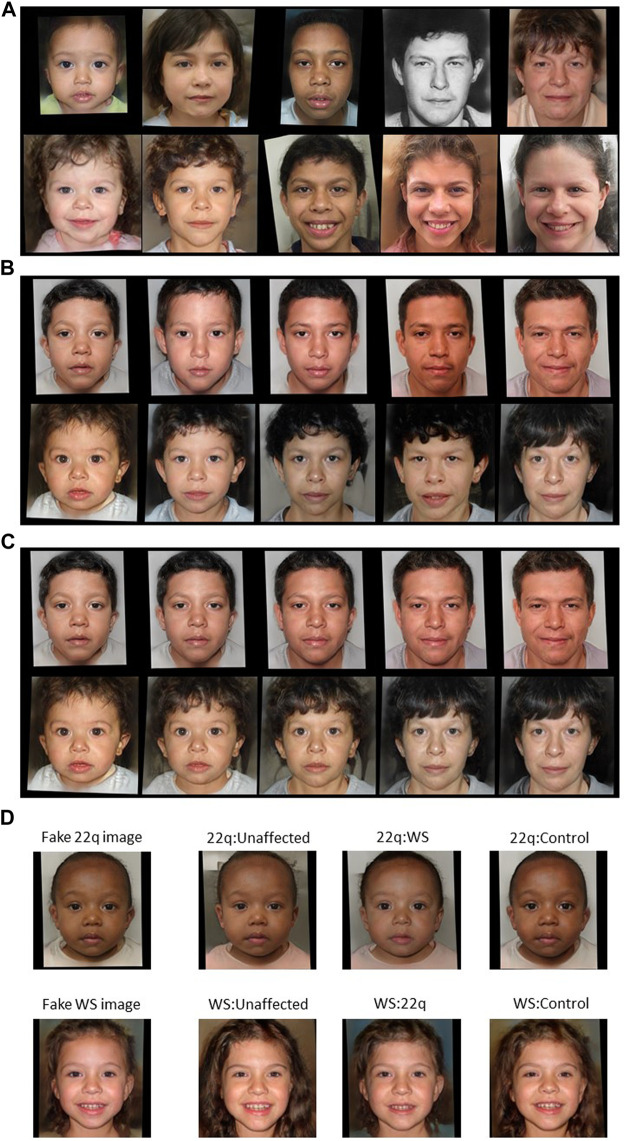
Examples of GAN fake images for 22q and WS. Type 1 fake images of **(A)** 22q (top row) and WS (bottom row) were generated with GAN and are all theoretically unique. For type 2 fake images **(B)** of 22q (top row) and WS (bottom row), general features, such as skin tone and hair color, are roughly preserved. For type 3 **(C)**, the generated images of 22q (top row) and WS (bottom row) look consistent at depicting the same “person” progressing through different age groups. Type 4 fake images **(D)** were created with blended facial characteristics of two disease labels. The main disease condition (22q or WS) represents 55% of the facial phenotype, and the added disease condition represents 45% of the facial phenotype. For example, WS:unaffected is a blend of 55% WS facial features and 45% unaffected facial features. Only the blended images were used for training, and the left most images are shown here as references.

For type 1, we generated a fake image *i* by concatenating the random vector *r*_*iad*_ with the label embedding *e*_*d*_ and *e*_*a*_, denoted as [*r*_*iad,*_ *e*_*a,*_ *e*_*d*_] and then passing this new vector to our GAN image generator. Each disease *d* and age group *a* combination has images generated from their own unique random vector *r*_*iad*_, so that all the fake images are theoretically unique (Figure 2A).

Type 2 images are generated by varying the age embedding *e*_*a*_, where a∈ {infant, child, adolescent, young adult, older adult}, while fixing the random vector *r*_*id*_ and the disease embedding *e*_*d*_ constant. The vector *r*_*id*_ is unique to the *i*th image having disease *d*. Following this, every disease has own unique images, but within the same disease the images at each age category have similar facial features such as skin tone and hair color (Figure 2B).

For type 3 images, we interpolated three equally spaced vectors between the age embedding e_*infant*_ and e_*older adult*_ *.* For a disease *d*, we generated five fake images from a random vector *r*_*id*_ by passing these five inputs, namely [*r*_*id*_ *, e*_*infant*_ *, e*_*d*_], [*r*_*id*_ *, 0.75e*_*infant*_ *+0.25e*_*older adult*_ *, e*_*d*_], [*r*_*id*_ *, 0.5e*_*infant*_ *+0.5e*_*older adult*_ *, e*_*d*_], [*r*_*id*_ *, 0.25e*_*infant*_ *+0.75e*_*older adult*_ *, e*_*d*_], and [*r*_*id*_ *, e*_*older adult*_ *, e*_*d*_] into the GAN generator. These images closely represent the same person affected with disease *d* at five different age groups (Figure 2C). There are additional potential approaches for depicting age progression, which we may explore in future studies (Or-El et al., 2020). Of note, the previous work (Gurovich et al., 2019; Porras et al., 2021) used different and/or additional age brackets, some of which may not involve sufficient numbers of images for robust analyses, at least in our datasets.

Type 4 images were generated like type 3 images; however, we reversed the roles of disease *d* and age label *a*. With a random vector *r*_*ia*_, we generated three fake images from the inputs: [*r*_*ia*_ *, e*_*a*_ *, ce*_*WS*_ *+* (*1-c*)*e*_*22q*_], [*r*_*ia*_ *, e*_*a*_ *, ce*_*WS*_ *+* (*1-c*)*e*_*control*_], and [*r*_*ia*_ *, e*_*a*_ *, ce*_*WS*_ *+* (*1-c*)*e*_*unaffected*_], where *c* is a predefined fraction between 0 and 1. These images represent a person at age *a* having facial characteristics of two different diseases (Figure 2D). The true labels for type 4 images are soft labels; for example, the image created from the vector [*r*_*ia*_ *, e*_*a*_ *, ce*_*WS*_ *+* (*1-c*)*e*_*22q*_] would have the label encoding [*c, 1-c, 0, 0*] instead of the traditional one-hot encoding. Here, the training loss function is still cross-entry but for soft label.

Next, we created four new larger datasets by combining partition *p* with each of the four fake image types. We then trained EfficientNet-B4 on each of these new larger datasets. For each type of new dataset, we created the ensemble predictor over all the data partitions following the approach mentioned in section *“Classifier.”*

### Attribution Analysis for Features in Different Age Groups

To visualize which facial features of an image the classifier considered to be important, we produced saliency maps using a window size 20 × 20 pixels and stride 10 × 10 pixels using the occlusion attribution method (Zeiler and Fergus, 2014). For a test image, we averaged the saliency maps of the classifiers in the ensemble predictor. We used the permutation test to measure how much the facial features identified by the classifiers differ with respect to age. Our Qualtrics surveys had 10 test images for each disease and age label combination. Conditioned on a disease and two age groups, we permuted the 20 images into two sets and repeated this permutation 100 times. Each time, we averaged the saliency map over the 10 images in each set and then retrieved the embedding of this average attribution via the EfficientNet-B4 trained on ImageNet. In each permutation, we computed the Euclidean distance between the embeddings of these two sets. If the observed Euclidean distance is smaller than 5% (or some other threshold) of the permutation values, then the two age groups of a specific disease were defined as not statistically different. That is, the key facial features identified by the classifier do not differ with respect to age.

## Results

### Classifier Accuracy

**FIGURE 3:**
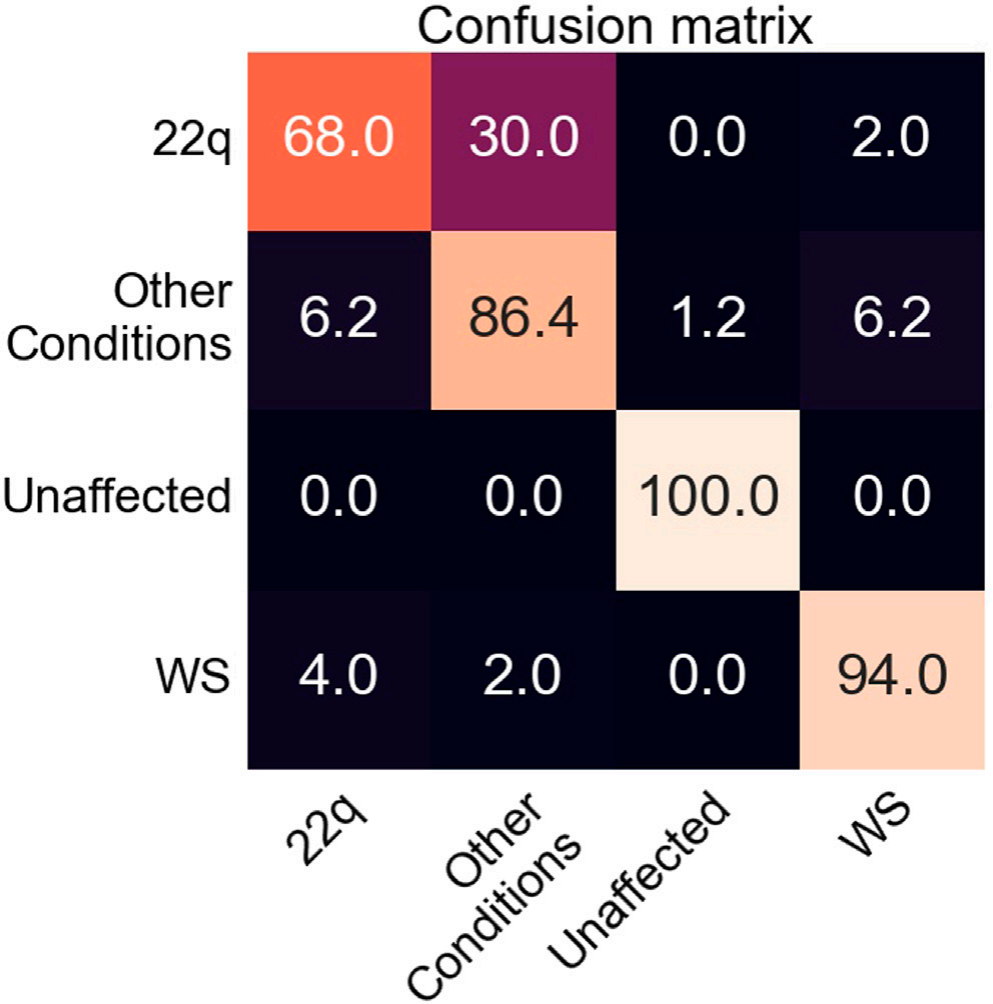
Confusion matrix of accuracy of the classifier trained on real images. Rows represent the correct label, while columns represent the label chosen by the classifier. The diagonal numbers represent the percent accuracy for each category (the percentage of images when the correct label was identified), while the off-diagonal numbers represent misclassification percentage ascribed to an incorrect category. Accuracy is based on 50 test images of WS, 50 of 22q, and 81 of other conditions.

Our classifier, which was trained on images of individuals with WS, 22q, other genetic conditions, and individuals who are presumably unaffected, correctly classified unique test images 68–100% of the time, with the lowest accuracy for 22q and the highest accuracy for unaffected individuals (Figure 3). While unaffected individuals are not misclassified as affected, the opposite is not typically true, presumably as some affected individuals may show only subtle features of the condition. A similar situation happens in clinical situations, where it can sometime be difficult to tell whether a person may be affected by a genetic condition and what that condition might be based on physical examination features (or other information). Classification accuracy of WS (86%) was the highest among the affected individuals we examined; our results suggest that these individuals often clearly display key findings (e.g., the dysmorphology or distinctive features affecting the eyes and mouth) compared to individuals with 22q, who are frequently misclassified (30%) as the control group (genetic or congenital conditions other than WS or 22q).

### Comparison to Clinical Geneticists

Clinical geneticists completed the survey(s) classifying images of individuals with WS versus other conditions and 22q versus other conditions. We intentionally used two separate surveys, based on preliminary testing, as we felt that WS and 22q would typically be considered a distinct condition by clinical geneticists and that it would be more meaningful to evaluate these conditions separately. The statistical differences between clinical geneticists and our model were measured via the paired *t*-test. As our model was trained with four labels, we took the highest prediction probability between WS and control for a test image in surveys containing WS and control images. The same idea applied to surveys containing 22q and their corresponding controls. Our model outperformed clinical geneticists by 15.5% (77.5 vs. 93%, *p* = 6.828e^−11^) for WS and by 22.7% (59.3 vs. 82%, *p* = 3.203e^−13^) for 22q.

To determine whether patient age affects accuracy, we determined the average accuracy for each age group (Table 1). On an average, the clinical geneticists had the most difficulty identifying infants affected with WS (67.3%) and the greatest accuracy with adolescents (80.7%). The clinical geneticists had the most difficulty classifying older adults with 22q (50.7%) and the greatest accuracy classifying adolescents (67.3%). Our classifier outperformed the clinical geneticists in all age groups (see Supplementary Table S5). However, we emphasize that because each age group has 10 images, performance differences may represent only a few images. Despite the small test size per condition and age bracket, our results suggest clinical diagnosis may be more difficult in some age groups.

**TABLE 1:** Average accuracy over all 10 test images in each age group. Each test image was viewed and classified by 15 clinical geneticists. Our classifier, either trained on real images alone or on both real and GAN age progression (age prog) images, obtains higher accuracy for each age group, except for the oldest 22q cohort. *P*-values in parentheses comparing the human against model were computed via the permutation test.

Age		22q			WS		
			Model			Model	
		Human	Real images	+ Age prog	Human	Real images	+ Age prog
Disease	Infant	0.54	0.7 (0.18)	0.8 (0.05)	0.673	1 (0)	1 (0
	Child	0.527	0.8 (0.04)	0.9 (0)	0.707	1 (0)	1 (0)
	Adolescent	0.673	0.7 (0.44)	0.8 (0.21)	0.0807	0.(0.24)	1 (0)
	Young adult	0.613	0.8 (0.10)	0.9 (0.01)	0.74	1 (0)	1 (0)
	Older adult	0.507	0.5 (0.50)	0.5 (0.50)	0.713	0.9 (0.09)	1 (0)
Other conditions	Infant	0.753	1 (0)	1 (0)	0.84	0.9 (0.35)	0.9 (0.35
	Child	0.6	0.9 (0)	0.9 (0)	0.767	0.8 (0.40)	0.8 (0.40)
	Adolescent	0.52	1 (0)	1 (0)	0.86	0.9 (0.39)	0.9 (0.39
	Young adult	0.6	0.9 (0.01)	1 (0)	0.787	0.9 (0.19)	0.9 (0.19
	Older adult	0.593	0.9 (0.01)	0.9 (0.01)	0.86	1 (0)	1 (0)

### Estimating Facial Differences Across Age Groups

**FIGURE 4:**
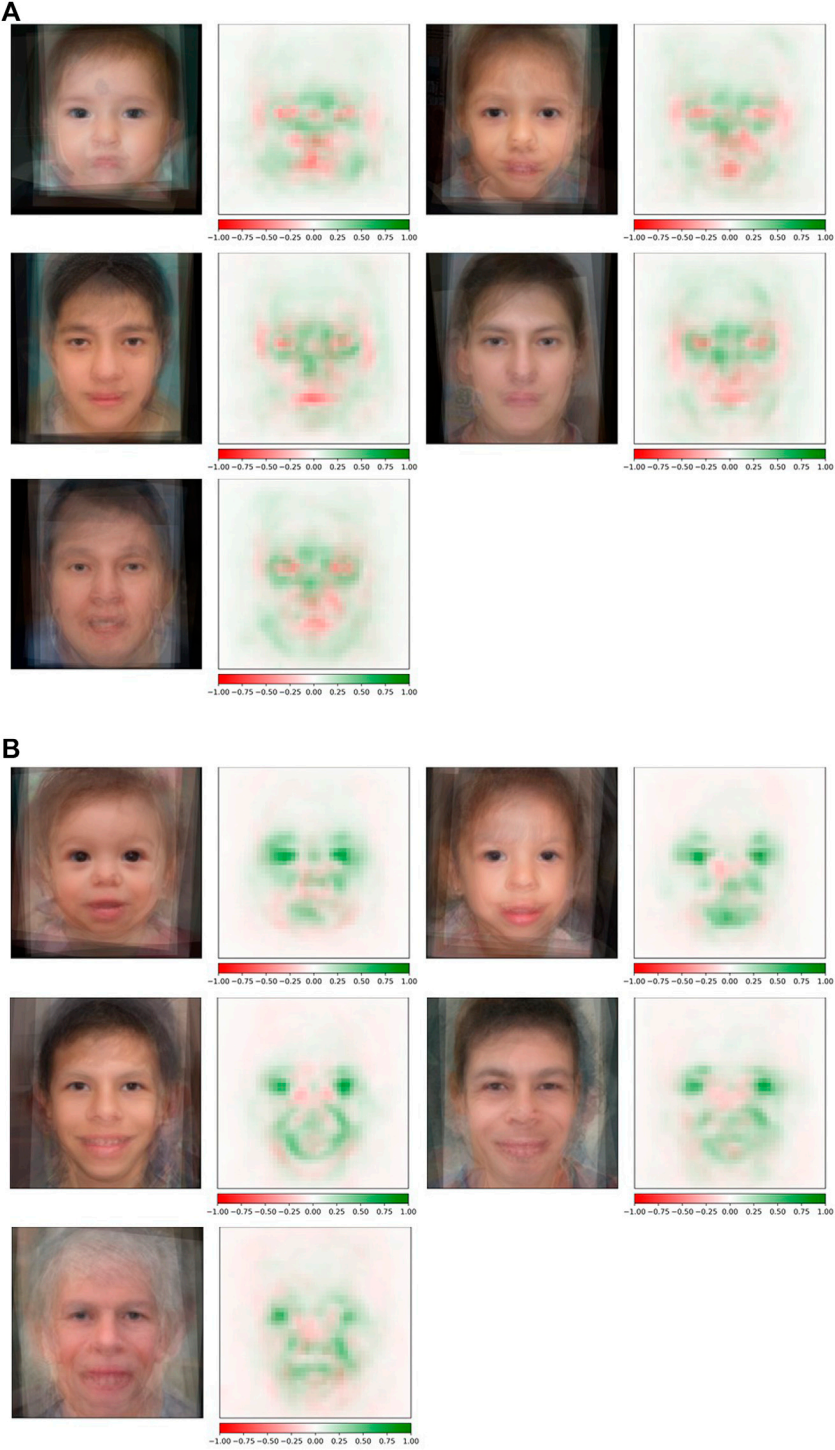
Occlusion analysis of 22q and WS facial images across the lifespan. Composite saliency maps were made by averaging over all the test images in each age group: infant, child, adolescent, young adult, and older adult (reading left to right) for both 22q **(A)** and WS **(B)**. Green indicates positive contribution, and red indicates negative contribution to the correct label. For 22q, specific regions of interest (e.g., periorbital regions, glabella, nasal bridge, and the mandible) subjectively appear to be consistently important at all ages analyzed. However, there appear to be some areas that are more specific for people in certain age groups, such as the areas superior to the lateral mandibular region in the youngest age group. Additionally, the periorbital region and nasal root appear to be more important in older age groups. For WS, facial features of interest across all age categories include the eyes (possibly due to the stellate iris or other important ocular features; we note that this pattern was not seen in people with 22q) and the mouth. Our composite WS images suggest that as aging progresses, the positive attribution present during infancy in the nasal root and periorbital region, as well as the eyes to some degree, decreases through older adulthood.

**FIGURE 5:**
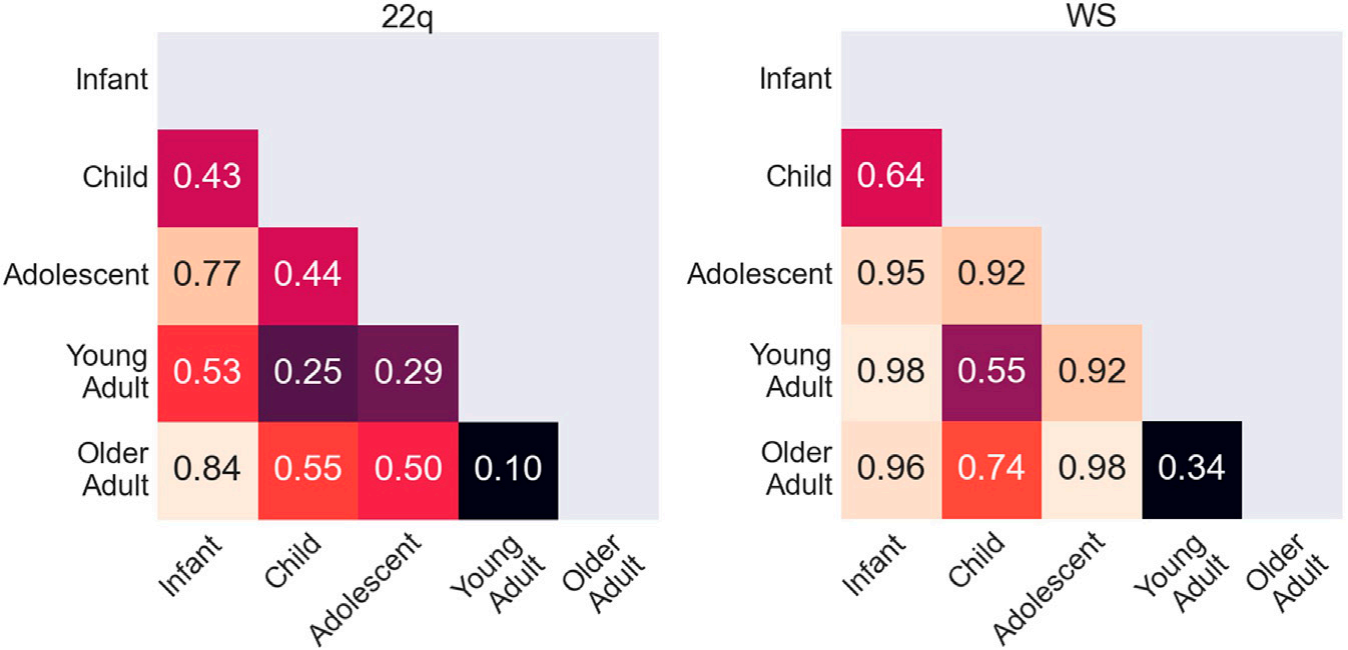
Quantitative comparison of key facial features during aging. Rank of the observed Euclidean distance (in fraction out of 100 permutations) between embeddings of the averages of occlusion analysis for two age groups. A small number indicates that key features identified by the neural network for two age groups are more statistically similar, whereas a larger number indicates that key features are more statistically different. Possible key facial feature differences across age categories are qualitatively explained in Figure 4.

Conditioned on a disease, we compared the saliency maps of the test images in different age groups based on the method in “*Attribution Analysis for Features in Different Age Groups*.*”* The composite images of saliency maps averaged over all the test images were generated for each age group. Figure 4 provides qualitative descriptions for the differences among the key facial features identified by the classifier for each age bracket. Figure 5 quantitatively compares these differences by measuring the Euclidean distances among the embeddings of these composite saliency maps. Assuming the standard 5% statistical significant threshold, there were significant differences among the five age brackets. For example, the observed distance between the embeddings of the 22q infant and child composite saliency maps ranks higher than 43 of the 100 permutation values (Figure 5). The greatest differences are seen for the infant and older adults in both WS and 22q. Compared to 22q, WS facial features identified by our model differ more with respect to age, which may show how facial features of a condition can be age-specific. Again, we emphasize that there can be confounding factors. For example, a person’s facial expression (e.g., whether a person is smiling) may explain why features of adolescent WS test images are different from those of the other age groups (Figure 5). Clinical geneticists may also rely on nonspecific facial clues (as well as other clinical manifestations) to classify syndromes and conditions. For example, high sociability and friendliness are common features of people with WS (Morris et al., 1993; Morris et al., 2020). While we did not intentionally select images based on facial expression (e.g., whether they were smiling or were not smiling), we found that more WS test images (60%) had a partial or full smile than other conditions (44%). Clinical geneticists were more likely to misclassify a Williams syndrome image if the image showed a person who was not smiling (58.3%) vs. smiling (82.4%) (see Supplementary Table S6). The presence or absence of a smile did not appear to impact classification of other conditions.

### Classifiers Trained on Real and GAN Images

Table 2 shows the accuracies of the classifiers trained on real images and different types of GAN images [see Supplementary Figure S2 for the corresponding confusion matrices]. Although the improvements are minimal, all four types of GAN images obtain slightly higher average accuracy than the base classifier, with type 3 GAN images (showing age progression for the same person) performing the best.

**TABLE 2:** Accuracy of the classifier trained on real images compared to classifiers trained on both real images and each of the four types of fake images. Column names unrelated, age related, age progression (age prog), and blended correspond to fake GAN images of type 1, 2, 3, and 4, respectively. The greatest improvement in accuracy was observed with the addition of age progression fake images.

	Real images	+ Unrelated	+ Age related	+ Age prog	+ Blended 55–45%	+ Blended 75–25%
22q	68	76	74	76	68	76
Controls	86.4	81.5	82.5	82.7	85.2	85.2
Unaffected	100	100	100	100	100	100
WS	94	96	96	100	96	96
Average	87.1	88.375	88.175	90.35	87.3	89.3

We also compared the classifier trained with type 3 GAN images against the clinical geneticists in each age bracket (Table 1). The cumulative improvements over humans are 95 vs. 77.5% (*p* = 1.846e^−11^) for WS and 87 vs. 59.3% (*p* = 1.703e^−15^) for 22q.

We suspect that type 3 GAN images improved the base classifier more because key facial features varied with respect to age (Figure 4, 5). By conditioning on the same person, we may capture more specific details of how a condition progresses with time.

## Discussion

The practice of medical genetics has shifted considerably in the last several decades. One major reason is the growing availability of high-throughput genetic/genomic testing, such as exome and genome sequencing. These testing methods allow more precise diagnosis and have changed the approach to phenotyping (Hennekam and Biesecker, 2012). However, access to these testing technologies is uneven, and it remains important to be able to quickly recognize patients who may be affected by certain conditions, especially those with near-term management implications (Solomon et al., 2013; Bick et al., 2021). For example, people with WS are prone to infantile electrolyte abnormalities and immunologic dysfunction, and people with 22q may be affected by endocrine, immunologic, cardiovascular, and other sequelae that require immediate attention (Campbell et al., 2018; Morris et al., 2020). Recognizing the likelihood of these conditions quickly—before the results of even the fastest genetic/genomic tests may be available—can be important for these and other conditions.

To provide examples of ways to bolster the standard diagnostic process as well as to build on the impressive findings of previous, related studies, (Gurovich et al., 2019; Duong et al., 2021b; Hsieh et al., 2021; Porras et al., 2021), we analyzed and provided a larger dataset of WS and 22q individuals (although these other studies contained a much larger total number of individuals having multiple other diseases). We also compared results for different ages of individuals. Our classifier outperformed clinical geneticists at identifying WS and 22q individuals by large margins (15.5 and 22.7%, respectively). This was consistently true for each age group.

We hypothesized that because geneticists overall often have more clinical experience with children, and as textbooks and the overall medical literature tend to focus more on pediatric presentations of congenital disorders, respondents would feel the most confident about diagnosis in younger age groups and would also perform best with images of younger patients. However, for WS, our results show that respondents’ accuracy did not correlate with their confidence level in diagnosing the conditions at various ages. For example, 46.7% (14/30) and 50% (15/30) of clinical geneticists surveyed reported that infants and older adults with WS are difficult to classify based on facial features, respectively, but the geneticists were able to classify these patients with similar accuracy to those of other ages (see Supplementary Table S7). This may imply other explanations. For example, clinicians may feel the most confident considering patients at ages they most often see in clinical practice, but this confidence may not be reflected in their performance. The features of WS may also be more pronounced with age such that clinicians can more readily recognize the condition in older patients, even when they have less real-life experience with patients at older ages. On the other hand, 60% (18/30) and 40% (12/30) of clinical geneticists, respectively, reported that infants and older adults affected with 22q are difficult to classify based on facial features, which aligns better with their performance in the survey we administered. There are again multiple explanations, but one possibility is that 22q may simply be a more subtle condition based on facial features, or that facial features in people with 22q do not become more obvious with age. Our saliency maps suggest that age-specific changes in key facial features exist in both 22q and WS. While saliency maps provide insights into the behavior of a neural network, these approaches have not been fully standardized or validated yet for the interpretation of medical data (Saporta et al., 2021). To explore these and other questions further, we plan to extend our analyses in the future to additional images and conditions, including by determining which particular features are objectively assessed by humans. This may help reveal underlying reasons for diagnostic patterns.

Intuitively, due to sample size difference, a classifier trained on fake and real images should outperform the one trained on just real images. Interestingly, this approach does not always improve the prediction outcome in previous works from other disciplines (Finlayson et al., 2018; Qin et al., 2020). Our results also showed that there was a small improvement with the incorporation of images (up to 3.25% accuracy gain). In the future, we plan to evaluate whether GAN images may be useful in other applications, for example, the generated images could help as educational tools. The GAN images could also be used to generate realistic images to obviate data sharing and privacy concerns. Along these lines, our results suggest areas of weakness that could be targeted for the generation of GANs, such as images of infants and older individuals, which could be used for medical training purposes. It would also be informative to conduct a meta-analysis on the existing literature across different disciplines to estimate the improvement of training GAN images and real images.

Our study has multiple limitations. First, our dataset is small compared to other datasets used for image recognition and may involve biases. Since collecting publicly free images of confirmed cases is challenging, we did not have balanced numbers of images for each condition and age bracket combination (Supplementary Table S3), and the types of images may have differed in certain categories. For example, we included some gray-scale images; having different numbers of these in some subsets could affect the color consistency for GAN-based transformation (see Supplementary Figure S3). However, the average age for each age grouping was consistent (see Supplementary Table S8). For example, the average age for the child age grouping was 5.54, 5.01, and 5.33 years for 22q, WS, and controls, respectively. Second, but also related to our sample size, we may have suboptimal grouping. For example, grouping all individuals older than a certain age into the oldest age group may have obscured differences within that group.

## Conclusion

Our contributions and findings can be summarized in the following four points.

First, we collected a dataset of publicly available WS and 22q images, which may be larger than others previously studied (Gurovich et al., 2019; Liu et al., 2021; Porras et al., 2021). Second, beyond the dataset, our approaches (and available code) may be used as subcomponents of other algorithms (Duong et al., 2021b). We trained a neural network classifier on our dataset (N = 1,894), which is still small compared to many other deep learning datasets, thus pushing the capability of the neural network model. Our classifier consistently outperformed clinical geneticists at recognizing individuals in the test set with these two syndromes for individuals in all five age brackets. Third, we show that key facial features (analyzed via saliency maps) identified by the classifier differ with respect to age. This type of approach is important for DL in biomedical contexts (DeGrave et al., 2021), including related to disease progression and other temporal factors. Fourth, there is a modest prediction accuracy increment by jointly training real images with different types of fake images created via GAN, in which including the fake images illustrating age progression for the same person yielded the best improvement.

Despite the rarity (and therefore lack of data availability) of many genetic conditions, neural networks have high potential in this area, due to both the ability to accurately categorize patients based on underlying molecular causes and the lack of trained experts throughout the world such that these tools could be highly valuable (Solomon, 2021). This area provides a ripe opportunity for patients, clinicians, researchers, and others to collaborate for the good of the impacted community. Privacy and data handling issues must be taken seriously; we hope that obstacles around data and code sharing can be addressed so as not to impose undue barriers for helping affected individuals and families.

## Data Availability

The datasets presented in this study can be found in online repositories. We make the versions of the publicly available images included in our analyses available (via CC0 license) for the purpose of reproducibility and research, with the assumption that these would only be used for purposes that would be considered fair use. These data were compiled to produce a new, derivative work, which we offer as a whole. The names of the repository/repositories and accession number(s) can be found via the link below: https://github.com/datduong/Classify-WS-22q-Img.
