# Supplementary material for: Neural Networks for Classification and Image Generation of Aging in Genetic Syndromes
Source: Front Genet. 2022 Apr 11;13:864092. doi: 10.3389/fgene.2022.864092 (PMC9035665; doi:10.3389/fgene.2022.864092)
Supplement: Supplementary file 1 [file Table1.docx]

| **Syndrome** | **Molecular Etiology** | **Inheritance pattern** | **Prevalence^a^** | **Common Features** | **Facial Features** | **Differential Diagnosis^b^** |
| --- | --- | --- | --- | --- | --- | --- |
| 22q11.2 Deletion Syndrome (other terms: DiGeorge syndrome, Velocardiofacial syndrome)(McDonald-McGinn et al., 1993) | Heterozygous deletion of 22q11.2 (1.5 to 2.54 Mb)  (Genes of interest in deleted region: *TBX1*; *DGCR8*; *CRKL*; *SNAP29*; and *PRODH*) | de novo (~90%), Autosomal Dominant | 1/4500 to 1/10,000 at birth | Congenital heart defects (primarily conotruncal malformations); palatal anomalies; distinctive (but often subtle) facial features; hypocalcemia; immune deficiency; learning difficulties | Hypertelorism epicanthal folds; prominent nasal root; short philtrum; micronathia; low-set ears(McDonald-McGinn et al., 2015) | Alagille syndrome; CHARGE syndrome;  Deletion 10p14-p14; Fetal alcohol spectrum; Goldenhar syndrome; Jacobsen syndrome; maternal diabetes, maternal retinoic acid exposure; Smith-Lemli-Opitz syndrome; VACTERL association |
| Williams Syndrome (other terms: Williams-Beuren syndrome)(Morris, 1993) | Heterozygous deletion of 7q11.23 (1.55 to 1.83 Mb)  (Genes of interest in deleted region: *ELN*; *LIMK1*; *GTF2I*; *STX1A*; *BAZ1B*; *CLIP2*; *GTF2IRD1*; *NCF1*) | de novo (93%), Autosomal Dominant | 1/7500 | Elastin arteriopathy(Collins, 2013) (supravalvular aortic stenosis, most common); distinctive facial features; intellectual disability; endocrine abnormalities; connective tissue abnormalities | Broad forehead; bitemporal narrowing; periorbital fullness; stellate irises; short, upturned nose; long philtrum; full lips(Morris, 2010) | 22q11.2 Deletion syndrome; Fetal alcohol spectrum; Kabuki syndrome; Noonan syndrome; Smith-Magenis syndrome |

**Supplementary Table 1:** Description of genetic conditions studied.

^a^Orphanet. Prevalence and incidence of rare diseases: Bibliographic data, January 2019, Number 01. 2019. https://www.orpha.net/orphacom/cahiers/docs/GB/Prevalence_of_rare_diseases_by_diseases.pdf. Accessed 1 November 2021.  Prevalence is given for the population unless otherwise stated.

^b^The differential diagnoses for these conditions are based on the overall phenotype, not just the facial features.

References

COLLINS, R. T., 2ND 2013. Cardiovascular disease in Williams syndrome. *Circulation,* 127**,** 2125-34.

MCDONALD-MCGINN, D. M., HAIN, H. S., EMANUEL, B. S. & ZACKAI, E. H. 1993. 22q11.2 Deletion Syndrome. *In:* ADAM, M. P., ARDINGER, H. H., PAGON, R. A., WALLACE, S. E., BEAN, L. J. H., MIRZAA, G. & AMEMIYA, A. (eds.) *GeneReviews((R)).* Seattle (WA).

MCDONALD-MCGINN, D. M., SULLIVAN, K. E., MARINO, B., PHILIP, N., SWILLEN, A., VORSTMAN, J. A., ZACKAI, E. H., EMANUEL, B. S., VERMEESCH, J. R., MORROW, B. E., SCAMBLER, P. J. & BASSETT, A. S. 2015. 22q11.2 deletion syndrome. *Nat Rev Dis Primers,* 1**,** 15071.

MORRIS, C. A. 1993. Williams Syndrome. *In:* ADAM, M. P., ARDINGER, H. H., PAGON, R. A., WALLACE, S. E., BEAN, L. J. H., MIRZAA, G. & AMEMIYA, A. (eds.) *GeneReviews((R)).* Seattle (WA).

MORRIS, C. A. 2010. Introduction: Williams syndrome. *Am J Med Genet C Semin Med Genet,* 154C**,** 203-8.
