# Supplementary material for: Neural Networks for Classification and Image Generation of Aging in Genetic Syndromes
Source: Front Genet. 2022 Apr 11;13:864092. doi: 10.3389/fgene.2022.864092 (PMC9035665; doi:10.3389/fgene.2022.864092)
Supplement: Supplementary file 2 [file Presentation1.PPTX]

## Slide 1
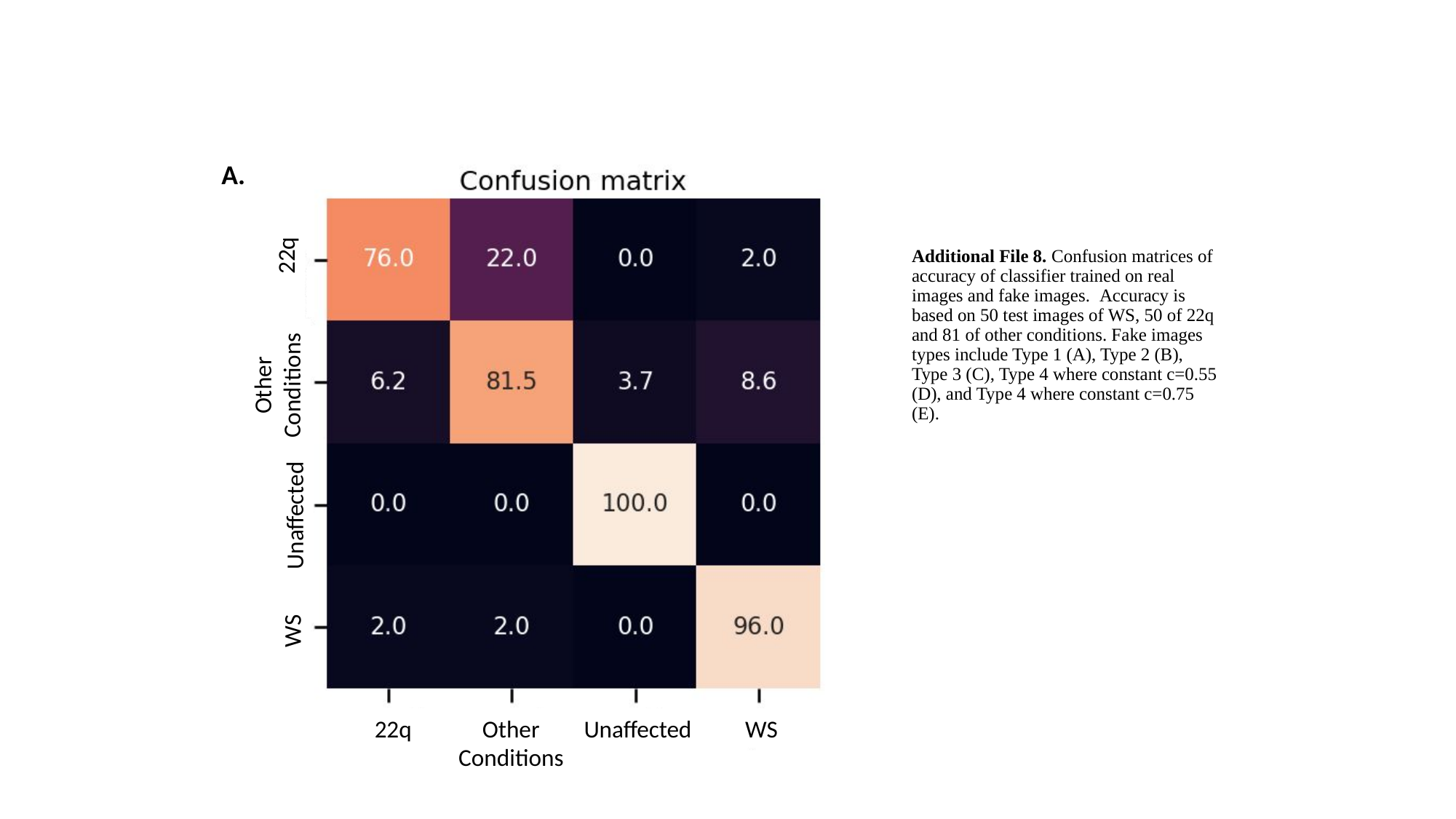

A.
22q
Additional File 8. Confusion matrices of accuracy of classifier trained on real images and fake images.  Accuracy is based on 50 test images of WS, 50 of 22q and 81 of other conditions. Fake images types include Type 1 (A), Type 2 (B), Type 3 (C), Type 4 where constant c=0.55 (D), and Type 4 where constant c=0.75 (E).
Other Conditions
  Unaffected
WS
22q
Other Conditions
WS
Unaffected

## Slide 2
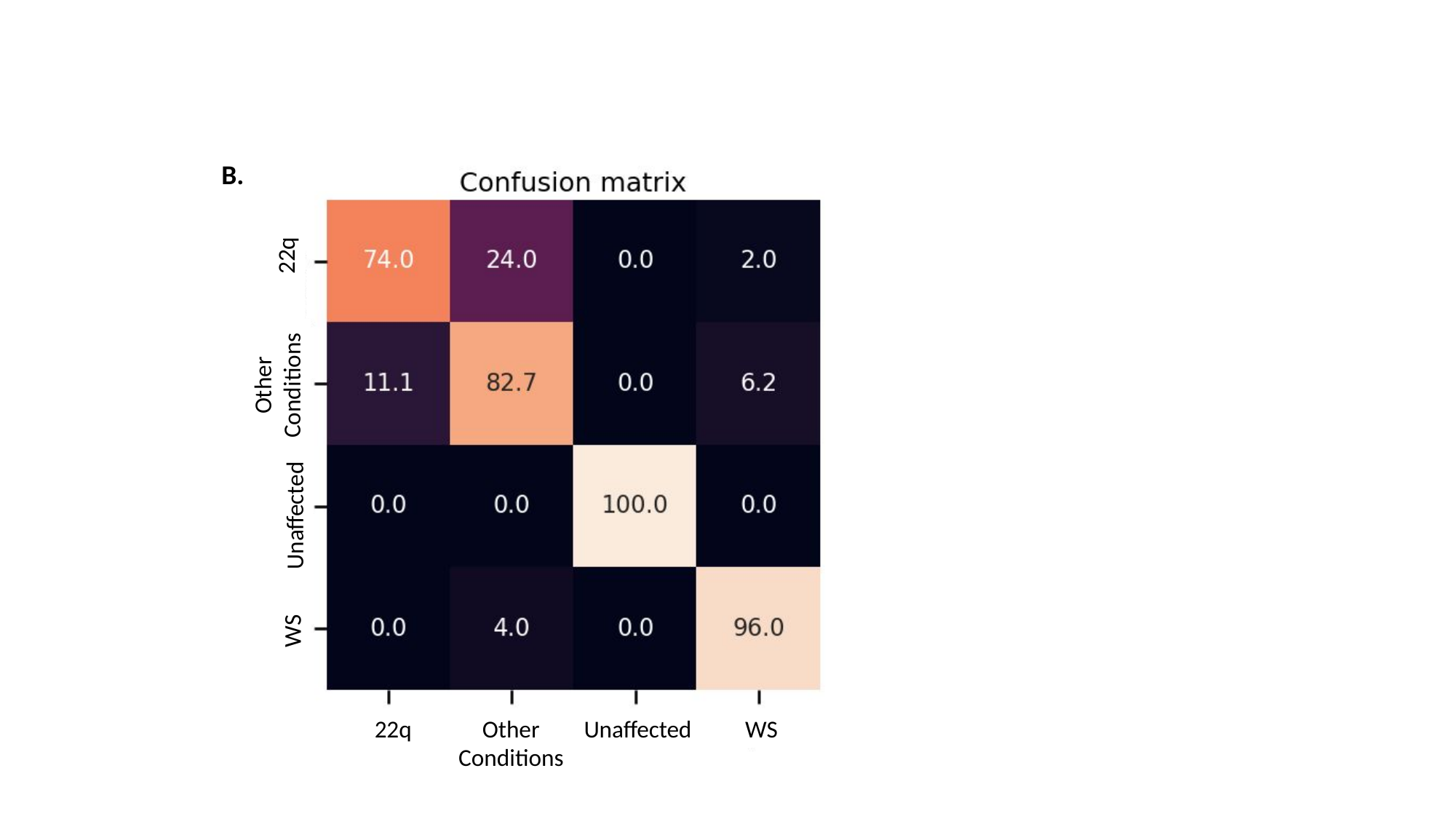

B.
22q
Other Conditions
  Unaffected
WS
22q
Other Conditions
WS
Unaffected

## Slide 3
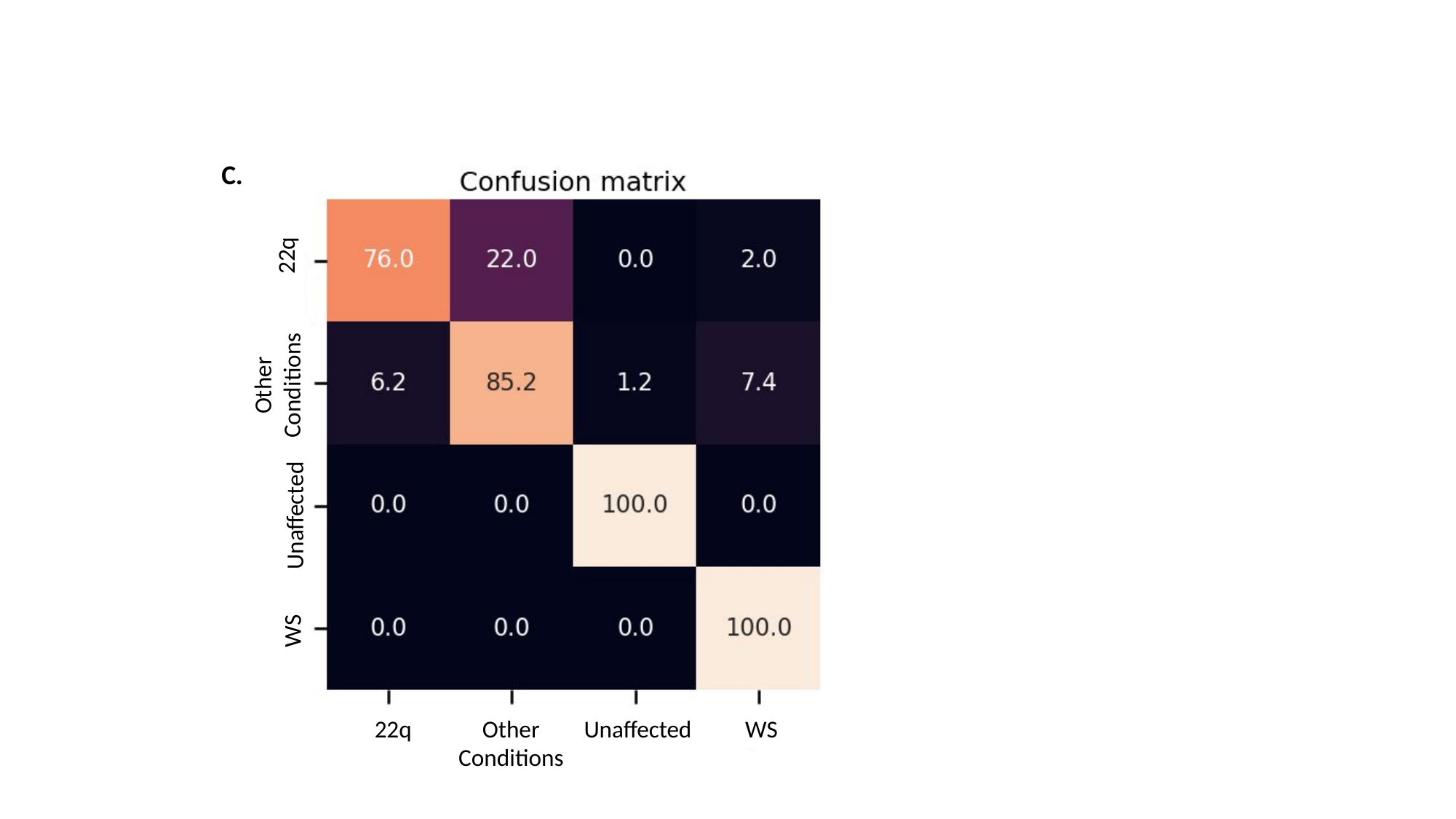

C.
22q
Other Conditions
  Unaffected
WS
22q
Other Conditions
WS
Unaffected

## Slide 4
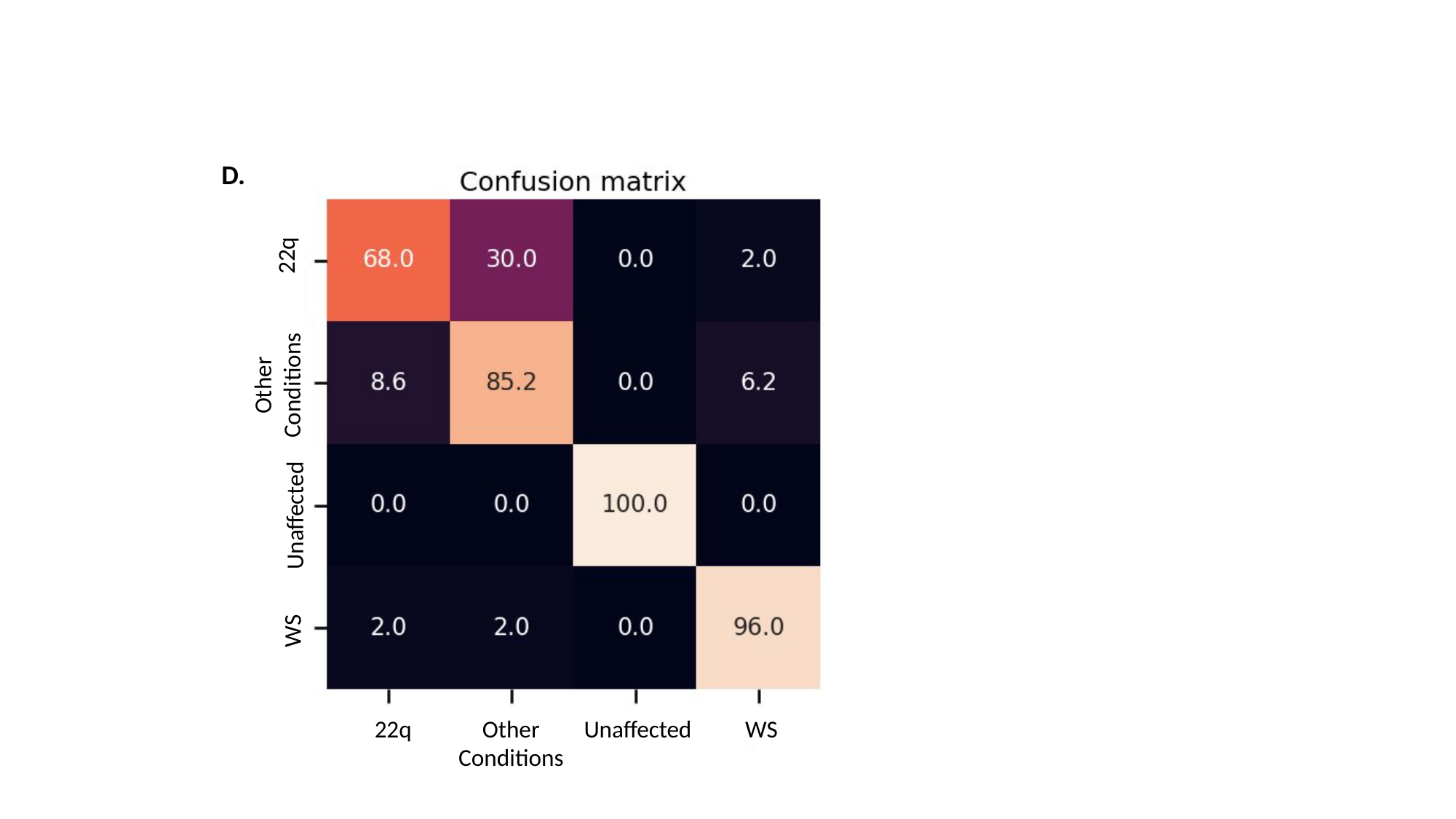

D.
22q
Other Conditions
  Unaffected
WS
22q
Other Conditions
WS
Unaffected

## Slide 5
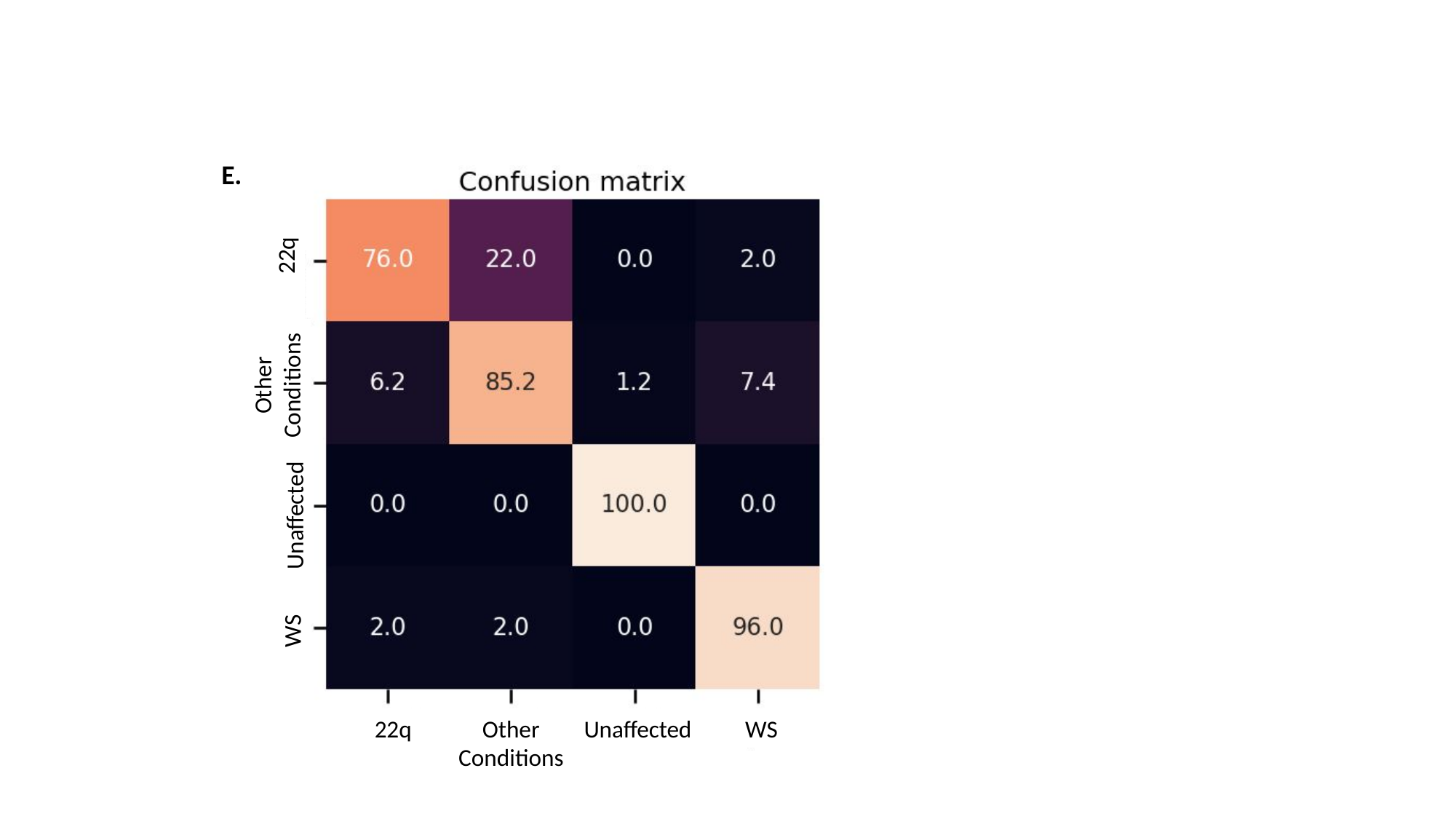

E.
22q
Other Conditions
  Unaffected
WS
22q
Other Conditions
WS
Unaffected
